# Agricultural intensification was associated with crop diversification in India (1947-2014)

**DOI:** 10.1371/journal.pone.0225555

**Published:** 2019-12-11

**Authors:** Jamey C. Smith, Aniruddha Ghosh, Robert J. Hijmans

**Affiliations:** Department of Environmental Science and Policy, University of California, Davis, California, United States of America; Instituto Nacional de Pesquisas da Amazonia, BRAZIL

## Abstract

Declines in agricultural biodiversity associated with modern farming practices may negatively affect the sustainability of agro-ecosystems, but formal knowledge of historical variation in spatio-temporal variation of agro-biodiversity is limited. We used time series of national (1947–2014) and district-level (1956–2008) crop distribution data for India to show that despite strong agricultural intensification after 1960, the average crop species diversity at the district level was stable, but increased at the country-level. While there was a decline in diversity in the major rice and wheat producing regions of northwestern India, associated with intensification of the production of these crops, diversity in western and southern India increased due to expansion of oilseeds and horticultural crops that replaced millet and sorghum. These opposite, but related, trends in crop-level diversity at the sub-national level partially canceled each other out at national level, but there nevertheless was a noticeable increase in overall crop diversity in India during this time period. Our results illustrate how patterns of change in crop diversity need to be considered at different levels of aggregation, and how a decrease in diversity associated with intensification and specialization in one area, may be associated with increased diversity elsewhere, and that support for intensive agriculture with relatively low crop diversity in some regions may be associated with an increase in crop diversity in other regions and at a higher level of aggregation.

## 1. Introduction

Biodiversity affects the structure and function of ecosystems and the services they provide [1]. It has been suggested that declines in agricultural biodiversity associated with modern farming practices may negatively affect the sustainability of agro-ecosystems, whereas increased biodiversity in agroecosystems may improve productivity and resiliency, and allow for hedging against crop failure [2, 3]. High agricultural biodiversity may also contribute to soil fertility [2], reduction of pests and diseases [4], yield stabilization [5] and a more varied and nutritious food supply [6, 7].

Agricultural biodiversity operates at various levels of organization (e.g., genes, species, crops and ecosystems) that are affected by different processes and may not all be changing in the same direction. There are substantial gaps in our understanding of how different levels of agricultural biodiversity change over space and time, what drives these changes, and how it affects agronomic, environmental, economic and social outcomes, particularly at scales larger than individual farms [8, 9]. For example, while modern crop varieties have replaced traditional varieties in many regions of the world, they have not in others, and in the absence of good baseline and contemporary surveys, there remains considerable debate about the extent of loss of genetic diversity in crop plants [10, 11, 12]. Likewise, decreases in landscape-level agricultural diversity associated with agricultural intensification (increased use of inputs per unit area), mechanization, specialization and increases in plot sizes have been described [13, 14] but it is not clear how general such observations are, and at what levels of spatial aggregation. For example, species-level crop diversity in the United States actually increased between 1870 and 1950 at both the state- and country-levels, during the period in which industrialized agriculture practices were being widely adopted [15], and species-level agricultural biodiversity in southern Quebec, Canada, was stable between 1911 and 1960 and increased after that [16].

Here, we analyze spatio-temporal patterns of change in crop diversity in India at the district, state, and national level of aggregation between 1947 and 2014, using a newly developed district level time series of crop distribution data. India is a good area to study the effect of intensification on diversity as it is a large and diverse country with an agricultural sector that has seen major changes during this era. After 1960, rice and wheat production intensified in many parts of the country, particularly in north-western India, with the “Green Revolution” of increased use of fertilizers and other agrochemical inputs, and the adoption of short straw varieties [17]. More recently, there has been an increase in the production of oilseed crops and of high-value horticultural crops for both export and domestic consumption (Joshi et al., 2004), as well as maize production (mostly for feed [18]), but generally speaking, food trade did not have a substantial impact on food availability during this period [19]. See Chakravarti [20] for a detailed account of the subnational variation in India’s agriculture and early adoption of “Green Revolution” technologies.

## 2. Methods

### 2.1 Data sources and pre-processing

District- and country-level crop area and production data for India from 1947 to 2014 were compiled. Data from multiple sources were combined into a new comprehensive database of crop distribution in India. Country level data covering 31 crops for 1947 to 1960 were obtained from a combination of Indian Statistical Abstracts [21] and the Food and Agriculture Organization of the United Nations (FAO) Statistical Yearbooks [22]. For 1961 to 2014, data covering 80 crops from FAOSTAT [23] were used.

India is currently divided into 29 states and 7 union territories (we refer to all these first level subdivisions as “states”) that are further subdivided into 722 districts. District level data were compiled from (i) the India Agriculture and Climate (IAC) data set, which covers 20 crops for 1956 to 1987 [24] and (ii) the International Crops Research Institute for the Semi-Arid Tropics (ICRISAT), which covers 24 crops for 19 states for the period from 1966 to 2011 [25]. ICRISAT data for 2009, 2010 and 2011 were incomplete and excluded from the analysis. Further details on the crops and years included in each dataset used are shown in the supplementary materials (S1 Table). Crop areas reported in both datasets for the overlapping years (1966–87) were very similar. The correlation coefficient for any crop was 0.98 or higher.

We combined the IAC and ICRISAT data by taking the average of the values for overlapping years. There have been many changes in the administrative boundaries of India during this time period. We used the district boundaries for the year 1966 as the base for the time-series. We matched the state-district combinations of ICRISAT with IAC data based on historical administrative atlas of India. Districts that were split during the time period covered in the analysis were aggregated (merged) to the original 1966 districts to create a consistent time-series. Missing values for crop-district combinations in which at least 10 values were available were imputed by temporal interpolation between the available values (for example, if the area of a crop was 100 ha in 1980, and 200 ha in 1982, but not reported for 1981, it was imputed to be 150 ha in 1981). Using the combination of datasets, time-series for 20 crops (1956–1965) or 24 crops (1966–2008) were generated for a total of 305 districts. State-level data were generated by aggregating district-level crop area and production, using current state boundaries. Crop yield at the district and state levels were calculated by dividing production by area.

We used the district level data to derive country level crop transition matrices to determine which crops replaced each other. Initial crop areas for each district were computed as the 5-year average for each crop from 1956–1960. Final crop areas for each district were computed as the 5-year average for each crop from 2004–2008. We made two matrices, one for the proportional and one for the absolute crop area, under the conservative assumption that the crop distribution within a district did not change. For example, if a district had 800 ha of rice during the initial time period, and 1000 ha of rice in the final, we assumed that 800 ha of rice did not change location, even though it is possible that rice was grown where there used to be another crop, and that previous rice area was in fact used for another crop.

### 2.2 Crop diversity

We computed crop species diversity (*D*) as the exponent of the Shannon diversity index (*H*) [26, 27]:

$$D=e^{H}$$

where,

$$H=-\sum_{i=0}^{n}p_{i}lnp_{i}$$

and *p_i_* is the proportion of the total crop area covered by crop *i*.

Diversity values (*D*) can be interpreted as the *effective* number of crop species, where a given value of *D* is equivalent to *D* species occupying an equal area [26].

All datasets contained different numbers of reported crops. For example, the district-level datasets report area for 20 or 24 major crops, whereas the country-level data from FAOSTAT (1961–2014) reports for 80 crops. Diversity values can be affected by the number of crops for which there is data available [15]. We therefore estimated what diversity would have been if data for the all unique crops in the country-level dataset had been reported at each level of spatial aggregation and for each year. Since diversity generally increases as a negative exponential of sample size [27], we used the following exponential regression model to fit the data:

$$D=a+b*e^{-c*x}$$

where *D* is the diversity value, *a*, *b*, and *c* are constants and *x* is the number of observed crops. Data to fit the model were obtained by subsampling the data available for each year / level of aggregation. Adjusted diversity values were computed by setting *x* to 83, i.e. the maximum number of unique crops observed in the country-level time-series from FAO. Except for the country level data, all diversity values reported in this paper are adjusted values.

For each district and state, a linear model was fit regressing diversity onto year. The slope of each model was used as a measure of change in diversity over time. Spatial variability in diversity, or *β*-diversity, was calculated for each year by dividing country-level diversity (*γ*-diversity) by the mean district-level diversity (*α*-diversity) for the same year [26]:

β=γ/α

*β*-diversity expresses the amount of dissimilarity between districts [27, 28]. That is, if all districts in a given year contain an equal proportion of all crops, *β*-diversity would be 1. Values greater than one indicate the degree to which districts are dissimilar.

### 2.3 Intensification & diversity

We investigated changes in diversity as a response to changes in agricultural intensification. Agricultural intensification is the increased use of land and/or inputs such as labor or fertilizer, normally leading to higher productivity. In this study we use crop yield as our measure of intensification. Cereals, particularly wheat and rice, were the crops most affected by the Green Revolution agricultural intensification in India. Two groups of models were fit with different crops used to calculate changes in intensification: 1) wheat only, and 2) rice, wheat, sorghum and millet.

To fit the models, we calculated the relative intensity (*RI*) for each district/year as follows:

$$RI=\frac{\sum_{i=1}^{n}p_{i}}{\sum_{i=1}^{n}a_{i}}/y_{max}$$

where *p*_*i*_ is the production of crop *i*, *a*_*i*_ is the area of cereal crop *i* (thus, the numerator of *RI* represents the weighted average of cereal yields for a given district/year), and *y*_*max*_ is the maximum area-weighted average cereal yield across all districts/years. Thus, the maximum value for *RI* is 1. We computed *RI* for each district at the beginning (*t*_*0*_, 1956–1960) and the end (*t*_*end*_, 2004–2008) of the time period for which we had district level data. Change in intensity (*ΔRI*) for each district was computed as the difference in intensity at *t*_*end*_ and *t*_*0*_.

For each of the two groups used to calculate *RI* (wheat only and the weighted average of wheat, rice, millet and sorghum), we fit linear models of diversity (*ΔD*) as a function of *ΔRI*, where *ΔD* was calculated as the difference in diversity at *t*_*end*_ (mean diversity from 2004–2008) and *t*_*0*_ (mean diversity from 1956–1960) for each district. For both groups, we fit models with *ΔRI* as the sole explanatory variable, and with an additional parameter for the initial proportion (at *t*_*0*_) of area in those crops included in the *ΔRI* calculation. This additional parameter was added to account for the areas where the crops included are relatively rare, and thus may not affect diversity much. We also fit models that included an interaction term between the *ΔRI* and initial area parameters. We used the Akaike Information Criterion (AIC) to select the most parsimonious final model.

After selecting a final model to explain change in diversity as a response to change in intensity, and the proportion of area at *t*_*0*_ planted to the cereal crops, we fit linear models to explain 1) the change in the proportional area of the cereal crops considered in response to change in intensity (*ΔRI*), and 2) the change in diversity (*ΔD*) as a response to a change in the proportional area planted to those crops. For each model, alternative versions with additional parameters for the proportion of area planted to the cereal crops at *t*_*0*_, and their interaction terms were compared. Final models were selected based on AIC scores.

While the above framework for explaining changes in diversity as a response to intensification worked well in those areas that were major rice and wheat producing regions at the beginning of the time-series, the models were not useful for millet and sorghum producing regions. To assess how diversity changed in the regions that were primarily millet and sorghum producers prior to the 1960s, we fit a linear model to the district-level data to show the change in diversity as a function of only the proportion of crop area planted to sorghum and millet at *t*_*0*_.

All data analysis was done with R [29], the R-packages used in this study are listed in S2 Table.

## 3. Results

### 3.1 Changes in crop area and yield

**Fig 1:**
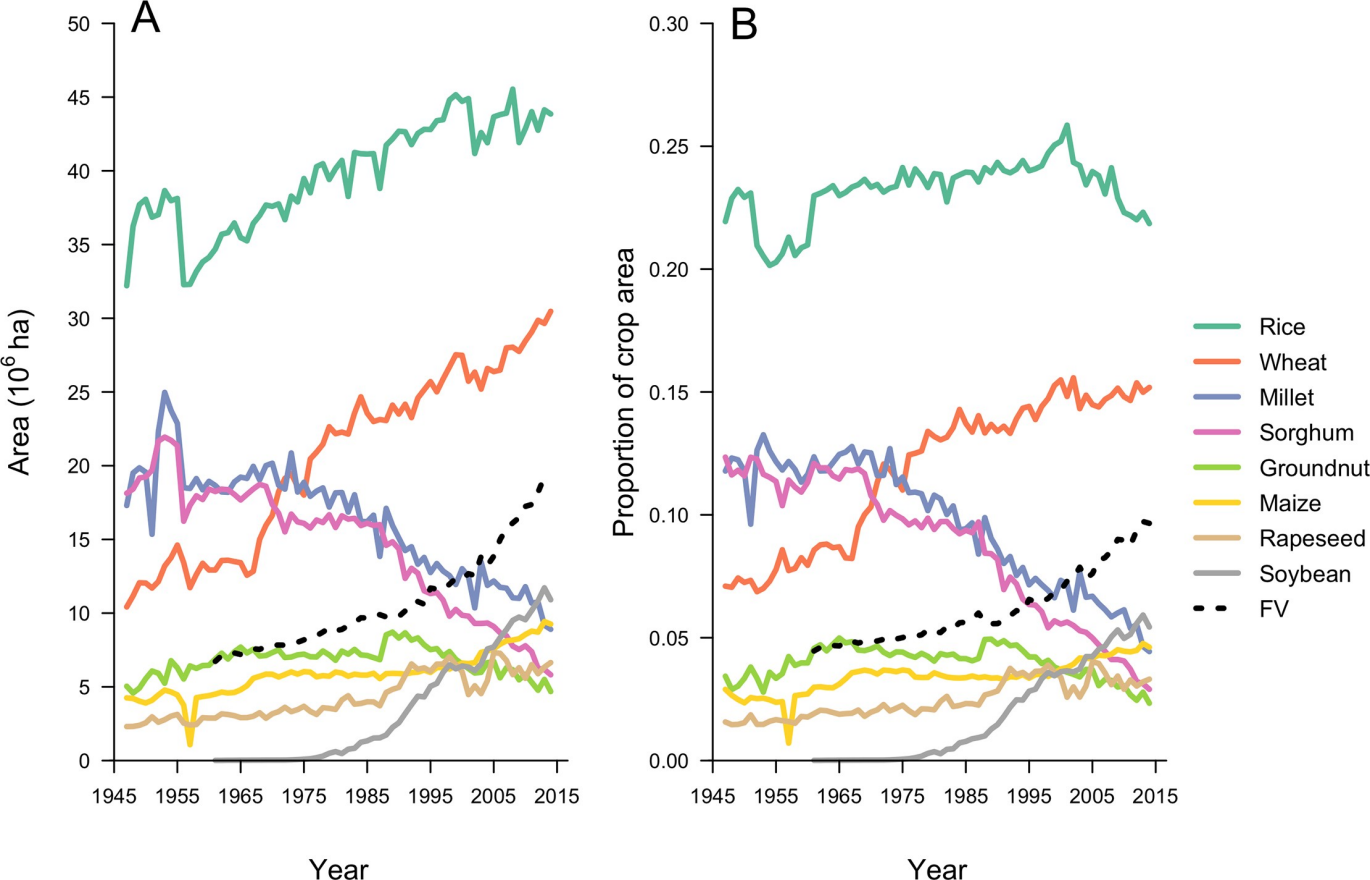
Area of 8 major crops and all fruits and vegetables (FV) in India between 1947 and 2014 (A) Crop area and (B) Proportion of crop area.

Crop area in India increased by 37%, from 147 million ha in 1947 to 201 million ha in 2014. Rice was the dominant crop throughout this time period; its area increased from 32 million ha in 1947 to 44 million ha in 2015 (Fig 1A). Wheat area nearly tripled during the same period from about 10 to 30 million ha and wheat is now the second most planted crop in India. Rapeseed and soybean expanded strongly after 1981 with rapeseed area leveling off at about 5.5 million ha after 1990 whereas soybean area continued to grow (Fig 1A). Up to 1975 there was very little soybean area in India; by 2014, there was more than 10 million ha. The rate of area increase for rice was 174,000 ha year^-1^ between 1947 and 2014, but that of wheat was much higher (299,000 ha year^-1^); After 1975, the rate of expansion for soybean was similar to that of wheat (234,000 ha year^-1^).

In contrast, area planted to millet and sorghum decreased strongly (Fig 1), with both crops losing about 2/3 of their area between 1947 and 2014. In 1947, wheat area was about half that of sorghum and that of millet, but around 1970 the area planted to these crops was very similar at approximately 18 million ha (Fig 1). The approximately 30 million ha planted to wheat in 2014 was more than millet and sorghum combined. The area planted with minor pulses was stable at around 14 million ha throughout the time period studied.

Area planted to all cereals increased sharply starting in the early 1960s, reaching a maximum of nearly 107 million ha in 1983 (S1 Fig). From the mid-1980s through the mid 1990s, absolute cereal area declined somewhat before leveling off to approximately 100 million ha after 1995 (S1 Fig). As a proportion of crop area, cereal area declined steadily from 63% of crop area in 1970, to 49% in 2014 (S1 Fig).

Rice area increased at a rate similar to the overall increase of crop area. Thus, the proportion of cropland planted with rice was stable between 1947 and 2014 at around 25%. During the same period, the proportion planted with wheat increased from 7 to 15%, while the percent of area planted to sorghum decreased from 12% to 3%. The proportion of area planted to all fruits and vegetables more than doubled from the early 1960s to 2014, increasing from about 4.5% in 1961 to nearly 10% in 2014.

Thus, the crop mix in India from 1947 to about 1975 was very different from what it is now. Before 1975 the dominant crop (rice) was followed by three large cereal crops (sorghum, millet and wheat) that each accounted for more than 10% of the crop area; together these cereals accounted for about 60% of the crop area. Rice is still as the dominant crop in terms of absolute and proportional area, followed by wheat at approximately 15% of crop area, and soybean with just over 5% (Fig 1). The area of many smaller crops (aggregated as Fruits and Vegetables) has increased sharply (Fig 1) and cereals now cover less than 50% of the total crop area (S1 Fig).

**Fig 2:**
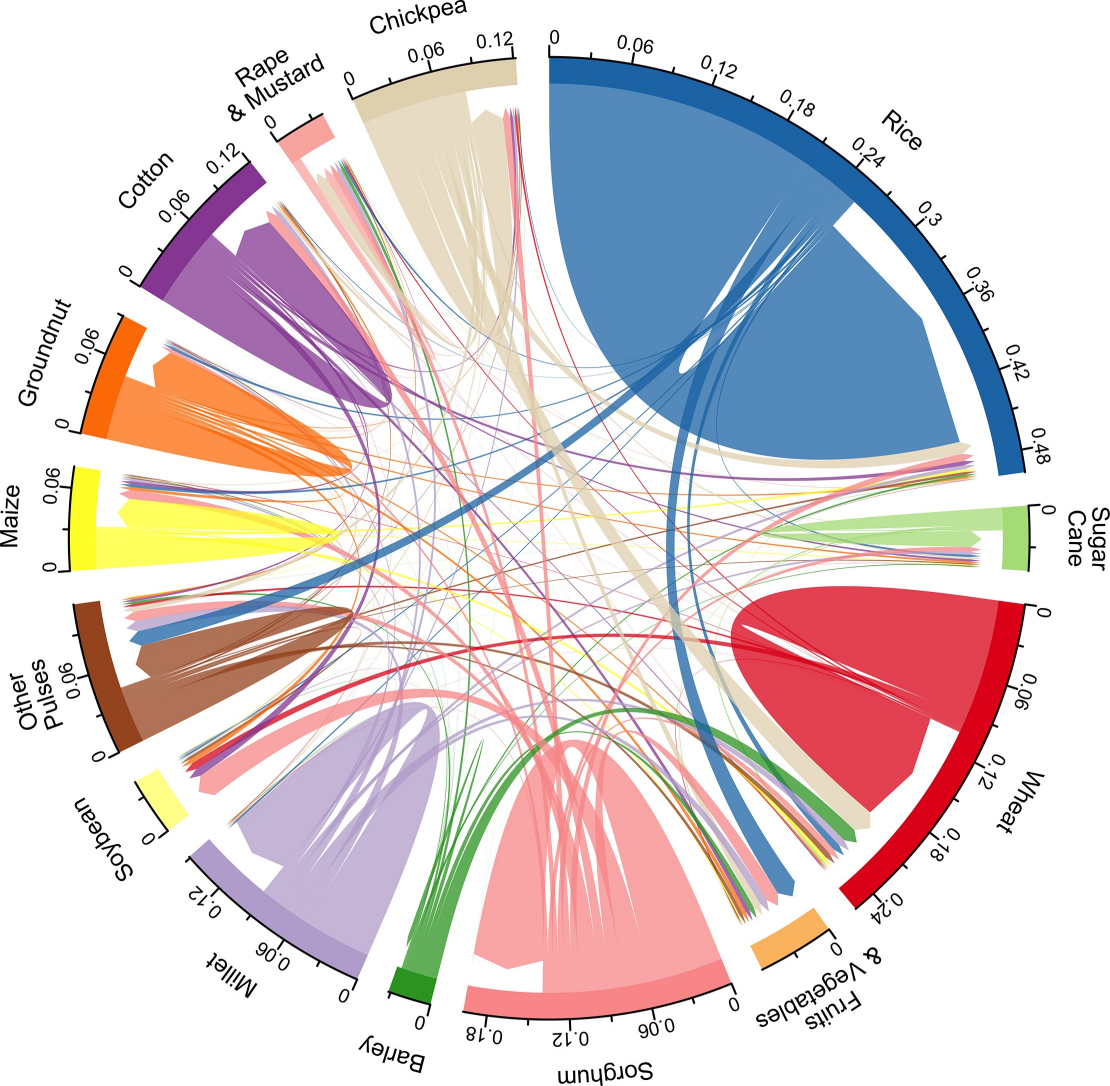
Crop turnover in India between 1956 and 2008 estimated from district level data. Values are the proportion of total crop area on a 1956–1960 (mean) basis. Arrows returning to the same crop indicate area that remained planted with that crop.

The vast majority of wheat and rice area remained in wheat and rice throughout the time period analyzed (Fig 2). Wheat expanded at the expense of many different crops, including chickpea, barley, and to a lesser degree millet and sorghum (Fig 2). The largest contributor to soybean area was land previously planted with sorghum, followed by cotton (Fig 2). Much of the sorghum, millet and barley area was replaced with rapeseed and mustard, as well as cotton, and fruits and vegetables. There was also substantial conversion from multiple crops to fruit and vegetables, and oilseeds (soybeans, rapeseed, and mustard) (Fig 2).

**Fig 3:**
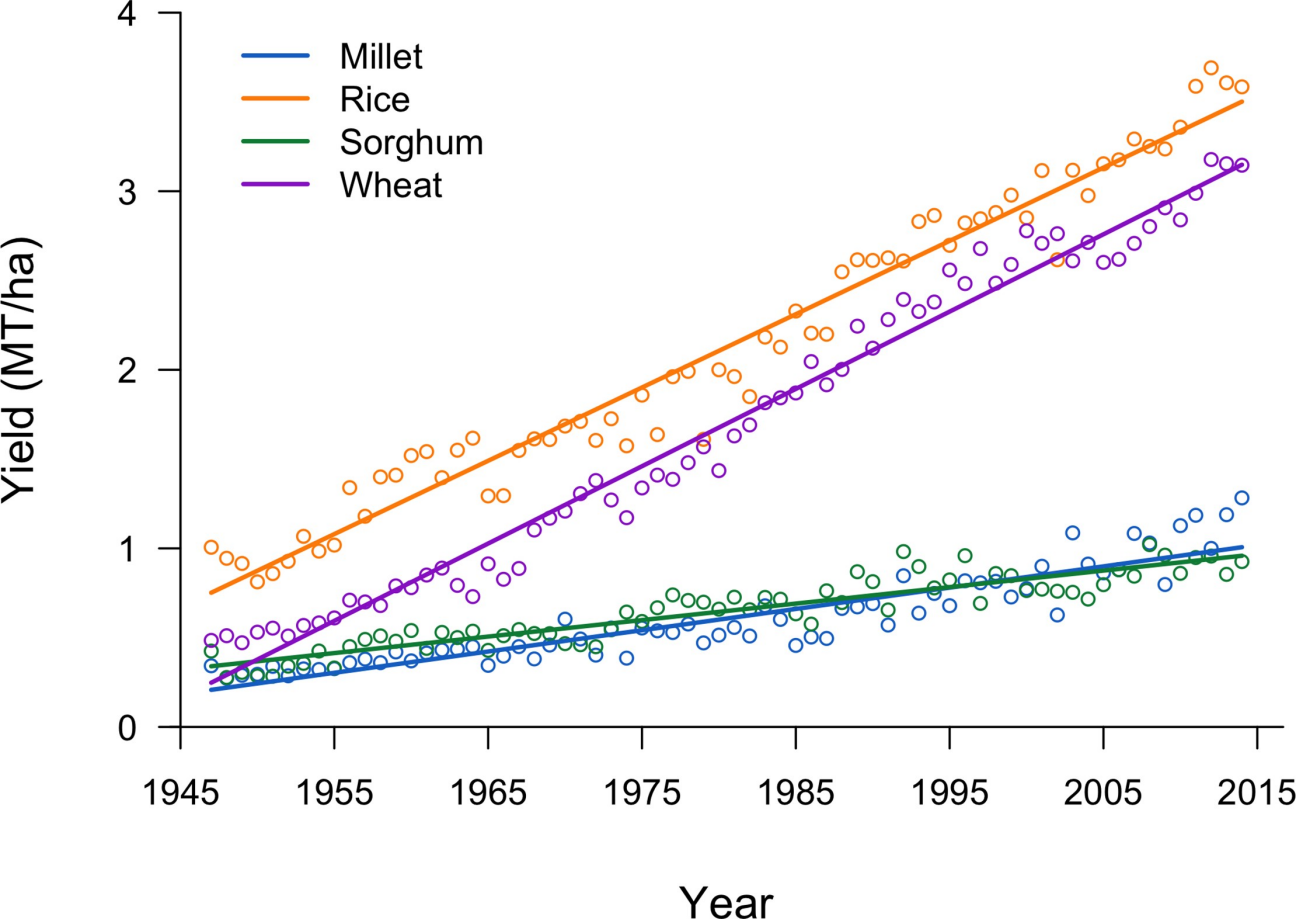
Grain yield of the four main cereal crops in India from 1947 to 2014, and linear trend lines.

Changes in yields were markedly different among the four dominant cereal crops at the beginning of the time-series (rice, wheat, millet, and sorghum). In 1947, yields of all four cereals were between 350 and 1000 kg ha^-1^ (Fig 3). By 2014, rice yields had increased to 3600 kg ha^-1^, and wheat yields had increased to 3100 kg ha^-1^, an average increase of 43 and 40 kg ha^-1^ year^-1^, respectively. Millet and sorghum yields change much less. In 2014, millet yields were 1300 kg ha^-1^, and sorghum yields were 920 kg ha^-1^, an average yearly increase of only 15 and 11 kg ha^-1^ year^-1^, respectively.

### 3.2 Changes in crop diversity

**Fig 4:**
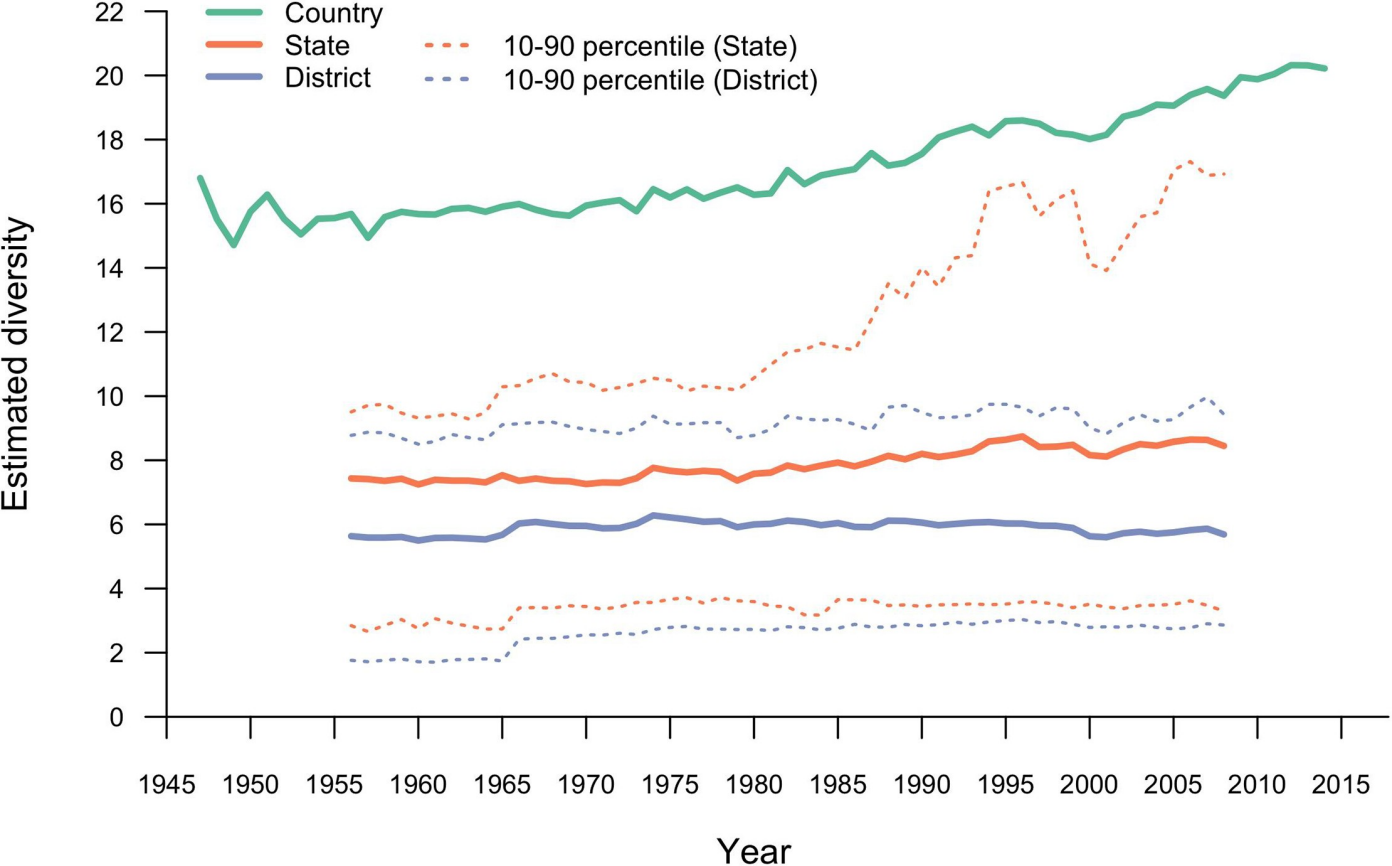
Crop diversity in India at the country, state, and district level. For the16 states and the 311 districts, the mean and the 10^th^ and 90^th^ percentiles are shown. Diversity is expressed as the “effective number of species”.

Mean district-level diversity was mostly stable at approximately 6 (that is, equivalent to 6 species at equal proportion) between 1956 and 1990, and declined to 5.7 in 2008 (Fig 3). Mean state-level diversity was stable from 1956 to 1974, with a mean of 7.4, then increased steadily to 8.4 in 2008 (Fig 4). The distribution of district-level diversity, as illustrated by the 10^th^ and 90^th^ percentile in Fig 4, tracked mean diversity. In contrast, the 90^th^ percentile state-level diversity increased at a higher rate than the mean, reaching a maximum of 17.3 in 2006. Country-level diversity was stable at about 15 between 1947 and 1960 and increased steadily in the years following, from 15.5 in 1961 to 20 in 2014 (Fig 4). Thus, while state-level diversity slowly increased after 1980, and district-level was mostly stable and declined a little, national level diversity increased at a higher rate, as districts became more dissimilar. This increase in dissimilarity is captured by the district level *β*-diversity for India, which oscillated around 2.7 until 1978 and increased steadily after that, reaching a level of 3.4 in 2008 (S2 Fig).

**Fig 5:**
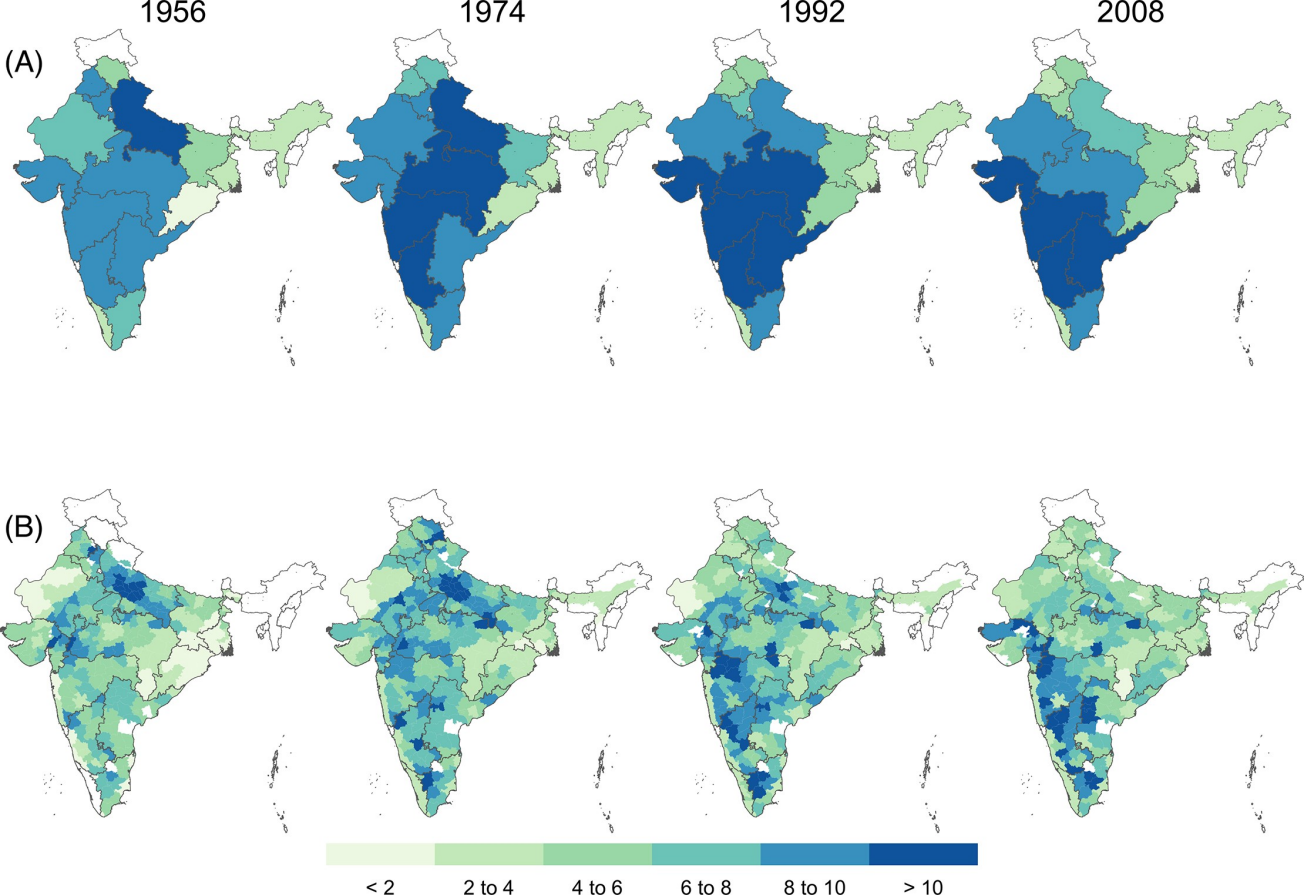
Crop diversity in India for in 1956, 1974, 1992 and 2008 at the (A) district and (B) state level. Data was not available for some districts and small states (white areas) for certain years. Diversity is expressed as the “effective number of species”.

**Fig 6:**
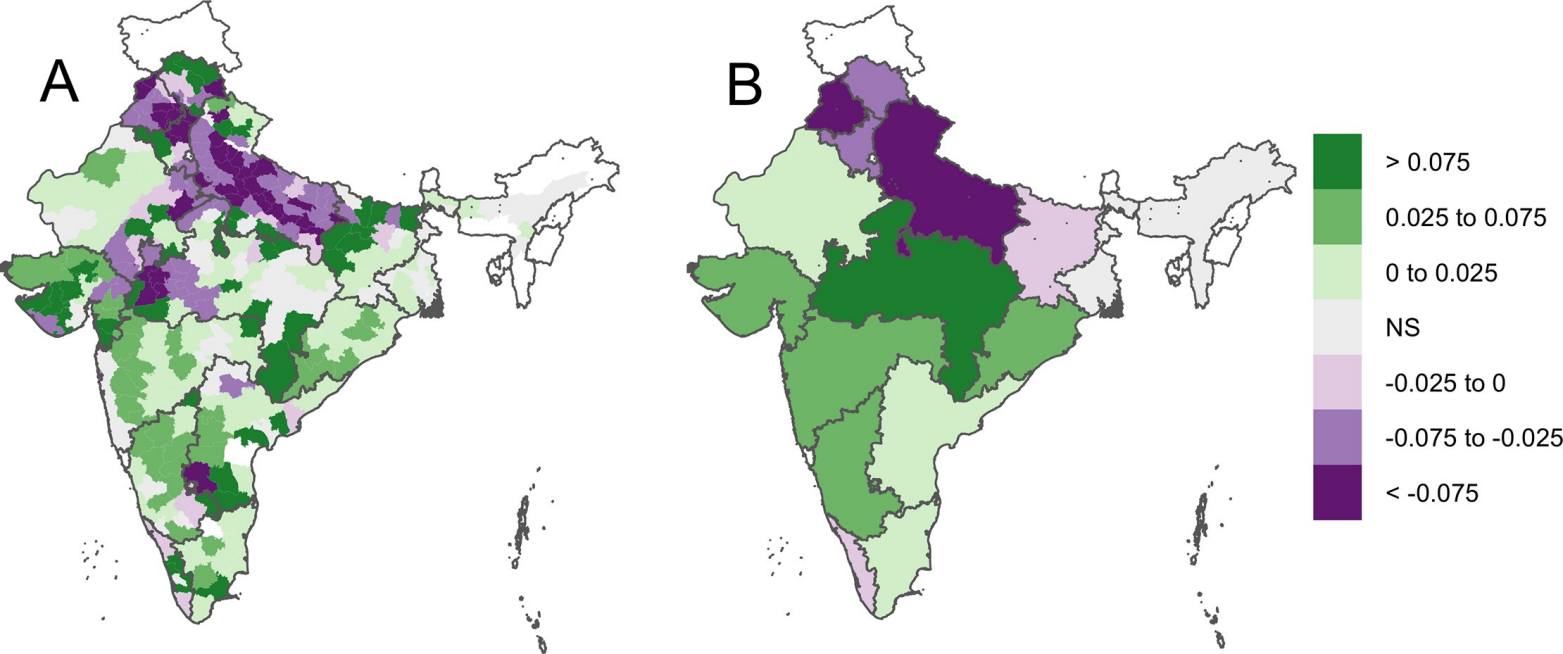
Slope coefficients of linear regression models of diversity as a function of year between 1956 and 2008 for (A) districts and (B) states in India. Regions with non-significant (P < 0.05) slope coefficients are labeled ‘NS’.

In 1956, the spatial distribution of crop diversity was relatively uniform across India, with marginally higher diversity in the northern states, and in Uttar Pradesh in particular, and lower diversity in the east (Fig 5). After that, crop diversity decreased sharply in northern India (Haryana, Punjab, and Uttar Pradesh states) as that region increasingly specialized in rice and wheat (Figs 5 and 6, and S3 and S4 Figs). By 1992, diversity in the wheat and rice producing northern region had declined noticeably, but it had increased in the south-western states of Gujarat, Maharashtra, and Karnataka (S5 Fig). Diversity was relatively high and stable, between 6 and 8 throughout the time period analyzed in several states, including Bihar, Madhya Pradesh, and Rajasthan (Fig 5). By 2008, most districts with high levels of crop diversity were in southwest India (Fig 5). Diversity increased in 50% of the districts and 41% of the states, and decreased in 34% of the districts and 27% of the states between 1956 and 2008 (P < 0.05). Diversity in the remaining 15% of districts and 23% of the states did not change (P > 0.05) (Fig 6).

### 3.3 Diversity and intensification

**Fig 7:**
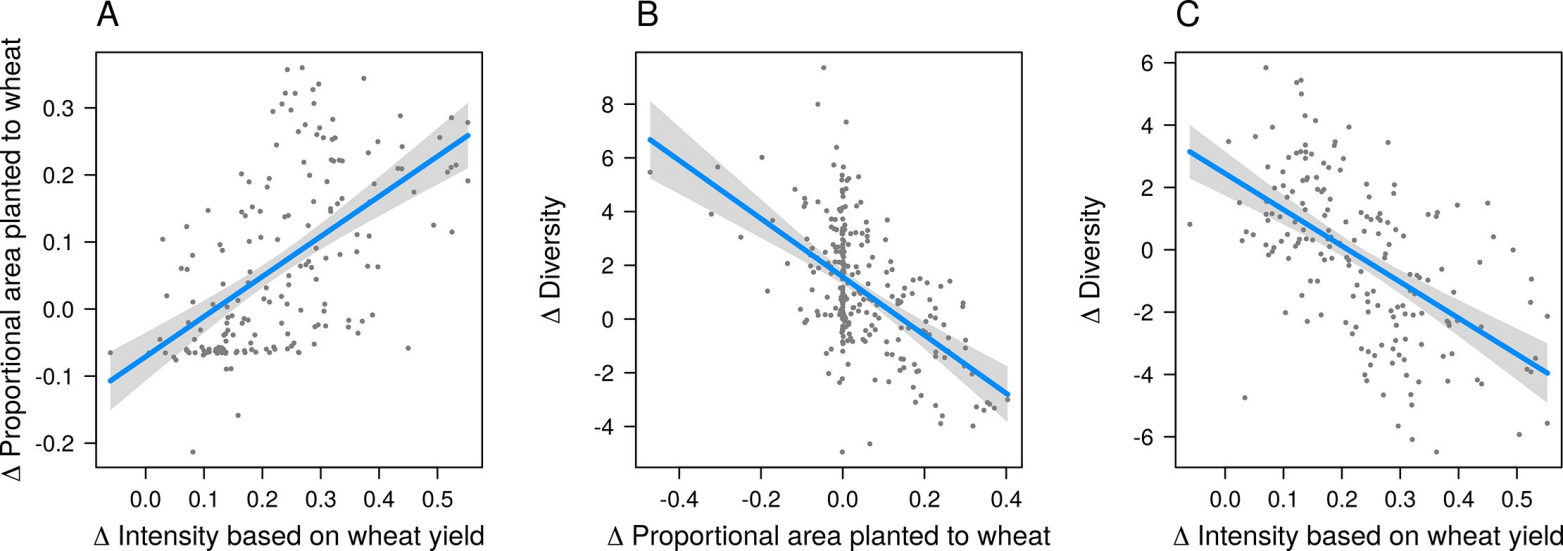
Regression models of the relation between agricultural intensification and changes in crop diversity in India between 1956 and 2008 at the district level. (A) Increase in the proportion of area planted to wheat as a response to wheat intensification. (B) Decline in crop diversity as a response to an increase in the proportion of area planted to wheat. (C) Decline in crop diversity in response to an increase in wheat yields. Other variables included in the linear models (not shown) were set to their median values.

We found a strong negative association between diversity and intensification (P < 0.001; Fig 7). The best fitting model included only wheat yield as an indicator of intensification along with an additional term for the initial proportion of crop area in wheat (Table 1). The “wheat only” model had the lowest AIC value and the highest R^2^ values of all models fit to the data (S1 Table). Additional models supported the notion that the wheat area increased in areas with most intensification of wheat, and that this was associated with a decrease in crop diversity (Fig 7, Table 1). The best fitting model of the change in the proportion of wheat area as a response to change in intensity (wheat yield) included an additional term for the initial proportion of area planted to wheat (Table 1). The best fitting model of change in diversity as a response to change in the proportion of wheat area included an additional term for the initial proportion of area planted to wheat, as well as an interaction term between the change in proportion of wheat area and the initial area planted to wheat (Table 1). The same trends were found to be significant in models using the weighted average of rice, wheat, millet, and sorghum cereal yields to calculate relative intensity, although model fit was generally lower than when using only wheat yield as an indicator of intensity (Table 1, S6 Fig).

**Table 1:** Parameters of best fitting linear regression models of changes in crop diversity and changes in area as a function of agricultural intensification (RI) based on wheat yield, or based on the area-weighted cereal (millet, sorghum, rice, wheat) yield and on area planted to the wheat or cereals. The last column shows the parameters of the best a model of changes in diversity and the area planted to the wheat or cereals.

RI Crop(s)	Variable	Response		
Wheat		Δ Diversity	Δ Proportional Area	Δ Diversity
	Intercept	3.0***	0.0	2.4***
	Δ RI	-11.6***	0.6***	-
	Area (*t*_*0*_)	-4.4***	-0.5***	-12.6***
	Δ Area	-	-	-10.0***
	Δ Area x Area (*t*_*0*_)	-	-	-13.3***
	R^2^	0.41	0.34	0.53
	Degrees of freedom	177	177	261
Cereals		Δ Diversity	Δ Proportional Area	Δ Diversity
	Intercept	0.7	0.0	-1.1*
	Δ RI	-8.5***	0.5***	-
	Area (*t*_*0*_)	2.5***	-0.4***	1.3*
	Δ Area	-	-	-6.0***
	Δ Area x Area (*t*_*0*_)	-	-	-
	R^2^	0.28	0.22	0.39
	Degrees of freedom	262	262	262

Statistical significance of explanatory variables is denoted using asterisks (***P < 0.001, **P < 0.01, *P < 0.05).

**Fig 8:**
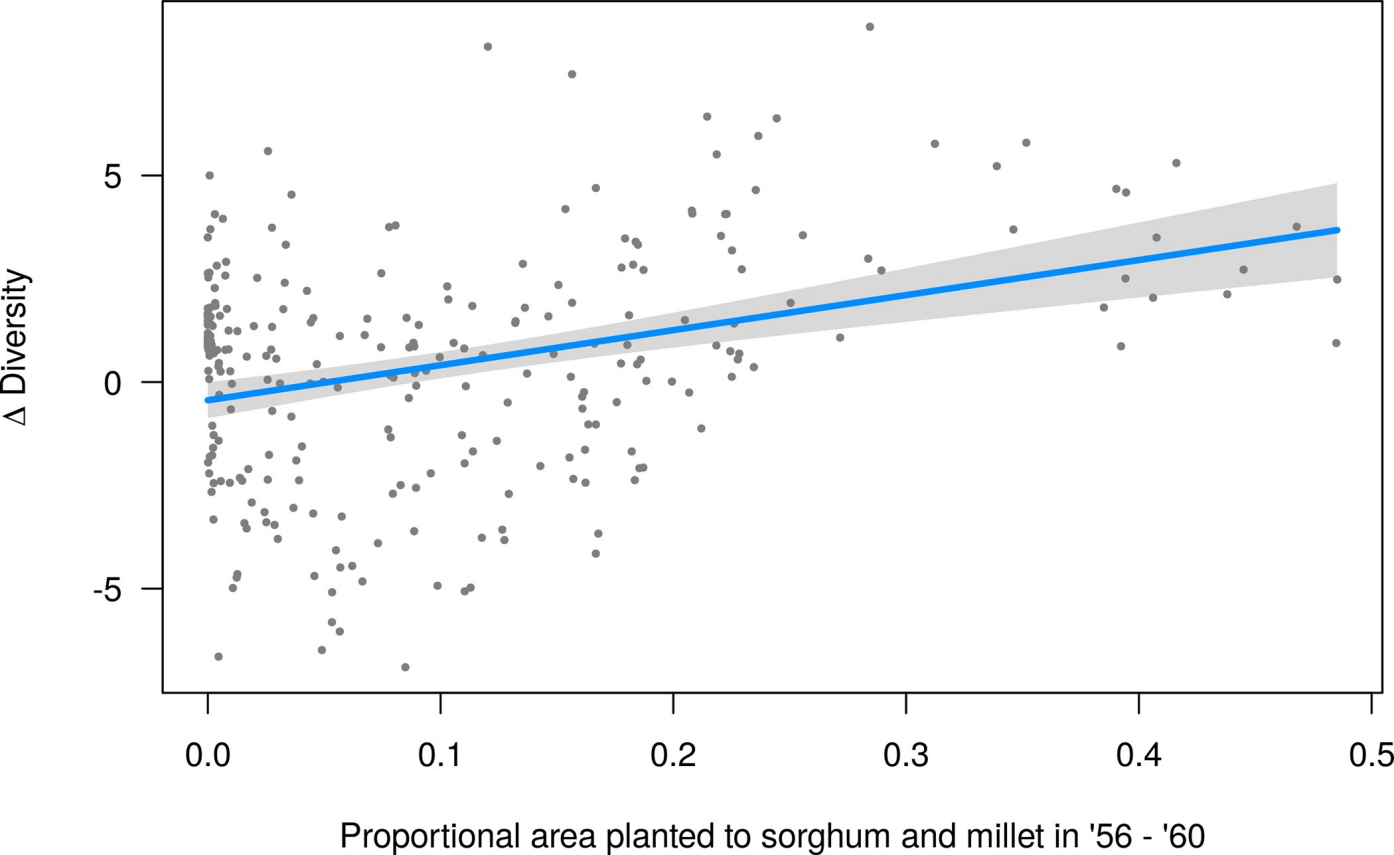
Increase in district-level crop diversity as a response to the area planted to sorghum and millet in 1956–1960.

We also found that diversity in those districts with higher proportions of area planted to sorghum and millet at *t*_*0*_ increased significantly (P < 0.001; R^2^ = 0.11) between 1956–2008 (Fig 8). When considered together with the state-level linear model slope coefficients (Fig 4), it is clear that area planted to millet and sorghum at the start of the times-series was a strong predictor of the direction of the change in a region’s crop diversity over the next 50 years. Karnataka, Gujarat, and Maharashtra states saw the most dramatic increases in crop diversity over the full time-series, with estimated increases in diversity of 0.23, 0.22, and 0.15 per year, respectively. These states were also major millet and sorghum producing regions in the mid- to late-1950’s. In 1956–1960, Gujarat was the 5^th^ largest sorghum (1.4 million ha) and 3^rd^ largest millet (1.6 million ha) producing state; Maharashtra was the largest sorghum (5.9 million ha) and 2^nd^ largest millet producing state (1.8 million ha); and Karnataka was the 2^nd^ largest sorghum (2.8 million ha) and 7^nd^ (0.5 million ha) largest millet producing state.

## 4. Discussion

Our results show that both spatial level of aggregation and extent are important when evaluating changes in crop diversity and its association with agricultural intensification. Crop diversity in India declined strongly over a 60 year period in certain regions, particularly in the rice- and wheat-producing districts of the Indo-Gangetic Plains, in which the effect of the Green Revolution was most dramatic [17]. However, crop diversity increased in other areas during the same time period, and at the national scale it increased steadily between 1960 and 2014. The decline in crop diversity since the mid 1960’s in India’s major cereal producing regions supports the idea that intensification of agricultural systems contributes to reductions in crop diversity. Intensification, as measured by relative wheat yields, was associated with increased specialization in rice and wheat production, particularly in those districts in which wheat and rice were the major crops at the start of the time-series, resulting in overall declines in crop diversity. Government policy during the same period, including input subsidies, price controls, and public investment in the creation of efficient grain distribution networks, likely contributed to increased price competitiveness of wheat and rice relative to sorghum and millet [30, 31]. Thus, simultaneous efficiency gains in both the production and distribution of wheat and rice may have contributed to an increased supply of these products over a larger area, making sorghum and millet less competitive. Together, these factors contributed to an increasingly specialized and concentrated cereal production in India following the introduction of Green Revolution technologies.

The simultaneous increase in diversity in other regions, particularly in the south west, shows another side of the relation between agricultural intensification and crop diversity. As the productivity gains in wheat (and to a lesser extent, rice) far outpaced that of millet and sorghum, the area planted to sorghum and millet declined, and their consumption declined more than that of other cereals [32, 33].

The productivity increases in wheat and rice producing regions, may have indirectly provided an incentive for farmers in other regions to diversify away from cereals. The regions that produced most millet and sorghum are generally warmer in winter and had less irrigation infrastructure and are therefore less amenable for (dry and cold season) wheat production. These regions benefited from policy support for crop diversification, particularly in those areas in which irrigation infrastructure was eventually expanded [30, 34].

Changes in crop diversity may have been further catalyzed by rising incomes and agricultural market reforms that resulted in decreased cereal consumption and increased demand for a broader range of agricultural goods including livestock products, fruits and vegetables, and oilseeds [32, 33]. Between 1983 and 2000, there were strong increases of per capita consumption of edible oils (~80%), vegetables (~45%), fruits (~175%), and meat, fish and eggs (~110%), while the per-capita consumption of pulses increased by about 8% [33]. Further research could assess the (sub-national) effects of intensification and diversification on dietary quality in India.

The steady increase in country-level crop diversity since 1965 can be largely attributed to the decline in a small number of important crops, especially sorghum and millet, and the simultaneous increase in area planted to oilseeds such as rapeseed, mustard, and especially soybean, and to the expansion of area planted to fruits and vegetables. The increase in country-level diversity following the decline of only a few significant crops, coupled with an increase in oilseed area is similar to crop diversity trends in the United States, where crop diversity increased after 1870 and peaked around 1960, largely due to a decline in the proportion of maize area and simultaneous increase in soybean area [15]. After 1960, area planted to several crops of intermediate size began to decline, resulting in a steady decline in country-level diversity in the second half of the twentieth century. It may be that crop diversity in India is on a similar, but lagged, trajectory as the United States, owing to the fact that many of the technologies enabling large-scale, intensified production of cereals and oilseeds in the United States were not introduced in India until more recently. However, it should be noted that the United States is a large food exporter, while India is not. Either way, crop diversity in India could decrease in the future if planted area of intermediate size crops such as sorghum, pulses, and millet continues to decline, and soybean production continues to increase.

Most research on crop diversity is at the field or landscape level (e.g., [13]), or focusses on intra-specific (genetic) diversity (e.g., [12]). Our study examines changes in crop diversity at higher levels of spatial aggregation and at the species level. We do not know the extent to which changes in crop diversity in India have led to changes in ecosystem services. Increases in diversity at a higher level of spatial aggregation do not necessarily correspond to increases at lower levels; and, as we have shown, trends in different regions may be in the opposite direction. A district may have diversified while at the same time fields and/or landscapes within that district have become less diverse. Further complicating an analysis of the ecological impacts associated with changes in crop diversity patterns in India is the fact that regional diversity increases were driven in part by agricultural expansion. It is therefore possible that, on the landscape level, total biological diversity certain regions decreased while crop diversity increased. It is also possible that crop diversity in rice and wheat producing regions, especially the Indo-Gangetic plains, may be lower if different metrics were used in the crop diversity calculation. Since the introduction of shorter duration varieties of wheat and rice, much of India’s cultivation of these crops (33% of rice and 42% of wheat by 1995) is in rotation [35]. That is, the same land is used to cultivate rice and wheat, at different times of the year. It can thus be important to consider different time scales in assessing diversity as well. We used an annual time scale and count two crops. But at any given time, there may only be one crop in all the fields.

The benefits of maintaining some level of crop diversity in agricultural systems are obvious and have been well established, but little is known about specific thresholds beyond which the ability of a system to provide certain ecosystem services declines. This is in part because the extent to which particular aspects of biodiversity affect agro-ecosystems depends on the biophysical context of the system, as well as spatial and temporal scale [36, 37, 38]. The country-level crop diversity increases in India can be considered beneficial. A more diverse agricultural sector allows for hedging against environmental or economic shock to specific crops [8, 39]. At the same time, however, cereal production is now highly concentrated in the Ingo-Gangetic plains, and geographic concentration can make it more vulnerable to shocks. Parts of this region are also strongly dependent on irrigation from depleting groundwater sources [40]. The extent to which an overall increase in India’s species-level crop diversity can be associated with any response, either positive or negative, in agroecosystem structure or function, and / or human health and wellbeing outcomes remains an important question.

## 5. Conclusion

Country-level crop diversity in India increased since the Green Revolution took off in the early 1960s. This national level trend is the aggregate of opposite trends at the sub-national level. The Indo-Gangetic Plains specialized further in wheat and rice production, leading to a decrease in diversity in these areas. This region is of fundamental importance for food security in India and a loss of diversity could make agriculture more sensitive to shocks such as the outbreak of a new disease. But it is important to consider the specific crops involved when assessing the effect of changes in crop diversity [41]. Low crop-diversity production systems of rice and wheat, the world’s most important food crops, have been common for a long time and appear to be relatively sustainable, especially in the case of flooded rice production. There are significant environmental problems in the region, most notably the depletion of ground water [42], but these concerns are a consequence of intensification, not of low diversity per se.

In the southwest of India, crop diversity increased as sorghum and millet were partly replaced with oilseeds and horticultural crops. In these regions, where crop production is predominantly rainfed, cropping systems have remained less intense, and crop diversity increased, driving an overall increase in country-level diversity following since the 1960s. These changes, at least in part a response to the specialization and intensification in the North, have contributed to somewhat higher national level crop diversity and provide the supply for more diverse diets.

Taken together our findings demonstrate the importance of considering various levels of aggregation and extent when examining patterns of crop diversity. Our results suggest that policy that promotes intensive agriculture with relatively low crop diversity in some regions, may lead to an overall increase in crop diversity, and perhaps dietary diversity, at a higher level of aggregation.

## Supporting information

S1 Table Spatial and temporal coverage, and crops included in the four datasets used. (PDF)

S2 Table R-packages used in the study. (PDF)

S1 Fig Total cereal area and the proportion of total crop area planted to cereals in India from 1961 to 2014. (PDF)

S2 Fig Crop district-level *β*-diversity (turnover) in India between 1956 and 2008. *β*-diversity was calculated for each year by dividing country-level diversity (*γ*-diversity) by the mean district-level diversity (*α*-diversity) for the same year: (*β* = *γ/α*).
(PDF)

S3 Fig Cereal production by district, as % of the national area, in India for select years between 1956 and 2008. (PDF)

S4 Fig (A). Proportional area of selected crops in India in 1956, 1982, and 2008. (B). Proportional area of selected crops in India in 1956, 1982, and 2008. (C). Proportional area of selected crops in India in 1956, 1982, and 2008.
(PDF)

S5 Fig Crop diversity in the 16 current states (1966 boundaries) included in the district level datasets for the period from 1956 to 2008. (PDF)

S6 Fig Regression models fit to district-level data to explain changes in crop diversity in India between 1956–2008 as a response to increased intensification. (A) Increase in the proportion of area planted to cereals as a response to increased intensity of cereals. (B) Decline in crop diversity as a response to an increase in the proportion of area planted to cereals. (C) Decline in crop diversity in response to an increase in cereal yields.
(PDF)
