# Supplementary material for: Agricultural intensification was associated with crop diversification in India (1947-2014)
Source: PLoS One. 2019 Dec 11;14(12):e0225555. doi: 10.1371/journal.pone.0225555 (PMC6905533; doi:10.1371/journal.pone.0225555)
Supplement: S1 Table — (PDF) [file pone.0225555.s001.pdf]

S1 Table. Spatial and temporal coverage, and crops included in the four datasets used.

Dataset	Spatial Resolution	Years covered	Number of crops	Crops
ICRISAT	District	1966-2008	24	Rice; Wheat; Sorghum; Pearl millet; Maize; Finger millet; Barley; Chickpea; Pigeonpea; Minor pulses; Groundnut; Sesame; Rape and mustard; Safflower; Castor; Linseed; Sunflower; Soybean; Sugarcane; Cotton; Fruits, Vegetables; Potatoes; Onion
IAC	District	1956-1987	20	Wheat; Rice; Sugarcane; Maize; Potatoes; Groundnut; Barley; Tobacco; Chickpea; Pigeonpea; Finger millet; Sesame; Rape and mustard; Pearl millet; Cotton; Sorghum; Minor pulses; Jute; Soybean; Sunflower
FAOYB/SA	Country	1947-1960	31	Rice; Sorghum; Millet; Maize; Wheat; Barley; Gram; Tur; Other pulses; Groundnuts; Castor seeds; Sesame; Rapeseed; Linseed; Cotton; Jute; Mesta; Tea; Coffee; Rubber; Coconut; Potatoes; Sugarcane; Black pepper; Chillies, dry, Ginger dry; Tobacco; Sweet potatoes; Cassava; Bananas; Hemp fiber
FAOSTAT	Country	1961-2014	80	Anise, badian, fennel, coriander; Apples; Apricots; Areca nuts; Bananas; Barley; Bastfibres, other; Beans, dry; Beans, green; Berries; Cabbages and other brassicas; Carrots and turnips; Cashew nuts, with shell; Cassava; Castor oil seed; Cauliflowers and broccoli; Cherries; Chick peas; Chilies and peppers, dry; Chilies and peppers, green; Cocoa, beans; Coconuts; Coffee, green; Cucumbers and gherkins; Eggplants (aubergines); Figs; Fruit, citrus; Fruit, fresh; Fruit, stone; Fruit, tropical fresh; Garlic; Ginger; Grapefruit (inc. pomelos); Grapes; Groundnuts; Jute; Lemons and limes; Lentils; Lettuce and chicory; Linseed; Maize; Mangoes, mangosteens, guavas; Melons, other (inc. cantaloupes); Millet; Nutmeg, mace and cardamoms; Oilseeds; Okra; Onions, dry; Oranges; Papayas; Peaches and nectarines; Pears; Peas, dry; Peas, green; Pepper (piper spp.); Pigeon peas; Pineapples; Plums and sloes; Potatoes; Pulses; Pumpkins, squash and gourds; Rapeseed; Rice; Rubber, natural; Safflower seed; Seed cotton; Sesame seed; Sorghum; Soybeans; Spices; Sugar cane; Sunflower seed; Sweet potatoes; Tea; Tobacco, unmanufactured; Tomatoes; Vegetables, fresh; Walnuts, with shell; Watermelons; Wheat
