# Supplementary material for: Agricultural intensification was associated with crop diversification in India (1947-2014)
Source: PLoS One. 2019 Dec 11;14(12):e0225555. doi: 10.1371/journal.pone.0225555 (PMC6905533; doi:10.1371/journal.pone.0225555)
Supplement: S2 Table — (PDF) [file pone.0225555.s002.pdf]

S2 Table. R-packages used in the study:

Baptiste A (2017). gridExtra: Miscellaneous Functions for "Grid" Graphics. R package version 2.3. https://CRAN.R-project.org/package=gridExtra

Bates D, Maechler M, Bolker B, Walker S (2015). Fitting Linear Mixed-Effects Models Using lme4. Journal of Statistical Software, 67(1), 1-48. doi:10.18637/jss.v067.i01.

Breheny P, Burchett W (2017). Visualization of Regression Models Using visreg. The R Journal, 9: 56-71.

Gu Z (2014) circlize implements and enhances circular visualization in R. Bioinformatics.

Guy JA (2019). migest: Methods for the Indirect Estimation of Bilateral Migration. R package version 1.8.0. https://CRAN.R-project.org/package=migest

Hijmans RJ (2019). raster: Geographic Data Analysis and Modeling. R package version 3.0-1. https://CRAN.R-project.org/package=raster

Lemon, J (2006) Plotrix: a package in the red light district of R. R-News, 6(4): 8-12.

Neuwirth E (2014). RColorBrewer: ColorBrewer Palettes. R package version 1.1-2.

Pebesma EJ, Bivand RS (2005). Classes and methods for spatial data in R. R News 5(2), https://cran.r-project.org/doc/Rnews/.

Wickham H (2017). tidyverse: Easily Install and Load the 'Tidyverse'. R package version 1.2.1. https://CRAN.R-project.org/package=tidyverse.
