# Supplementary figures and images for: Agricultural intensification was associated with crop diversification in India (1947-2014)
Source: PLoS One. 2019 Dec 11;14(12):e0225555. doi: 10.1371/journal.pone.0225555 (PMC6905533; doi:10.1371/journal.pone.0225555)

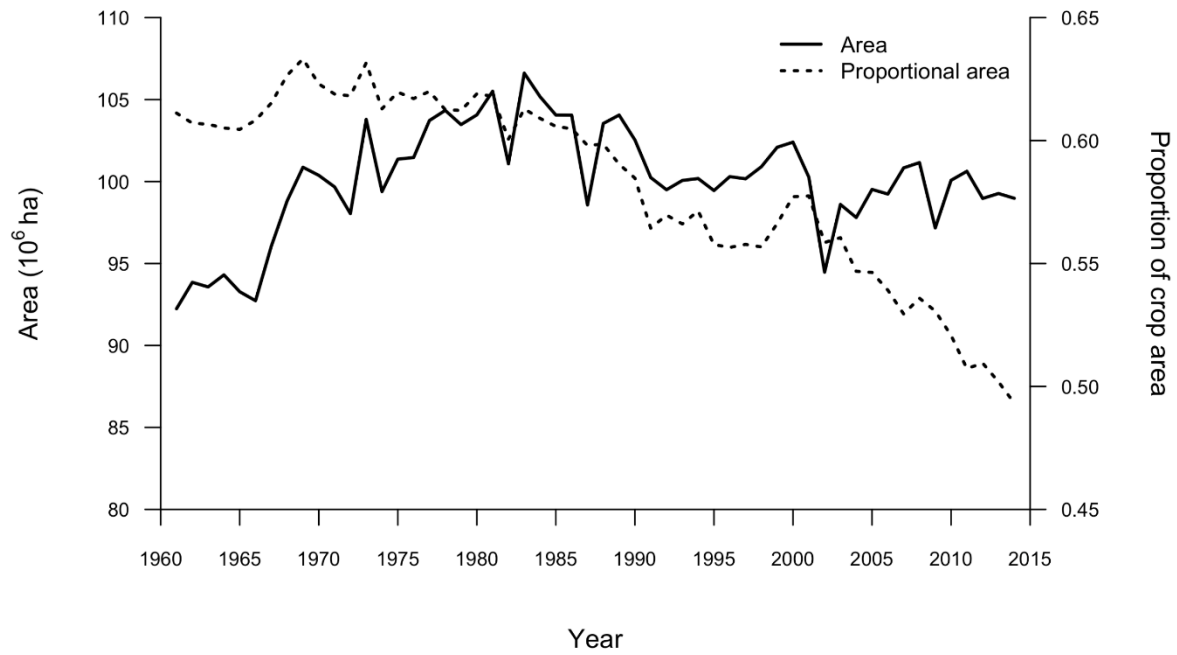

**S1 Fig.** Total cereal area and the proportion of total crop area planted to cereals in India from 1961 to 2014.

Supplement: S1 Fig — (PDF) [file pone.0225555.s003.pdf]
