# Supplementary material for: Agricultural intensification was associated with crop diversification in India (1947-2014)
Source: PLoS One. 2019 Dec 11;14(12):e0225555. doi: 10.1371/journal.pone.0225555 (PMC6905533; doi:10.1371/journal.pone.0225555)
Supplement: S2 Fig — β-diversity was calculated for each year by dividing country-level diversity (γ-diversity) by the mean district-level diversity (α-diversity) for the same year: (β = γ/α). (PDF) [file pone.0225555.s004.pdf]

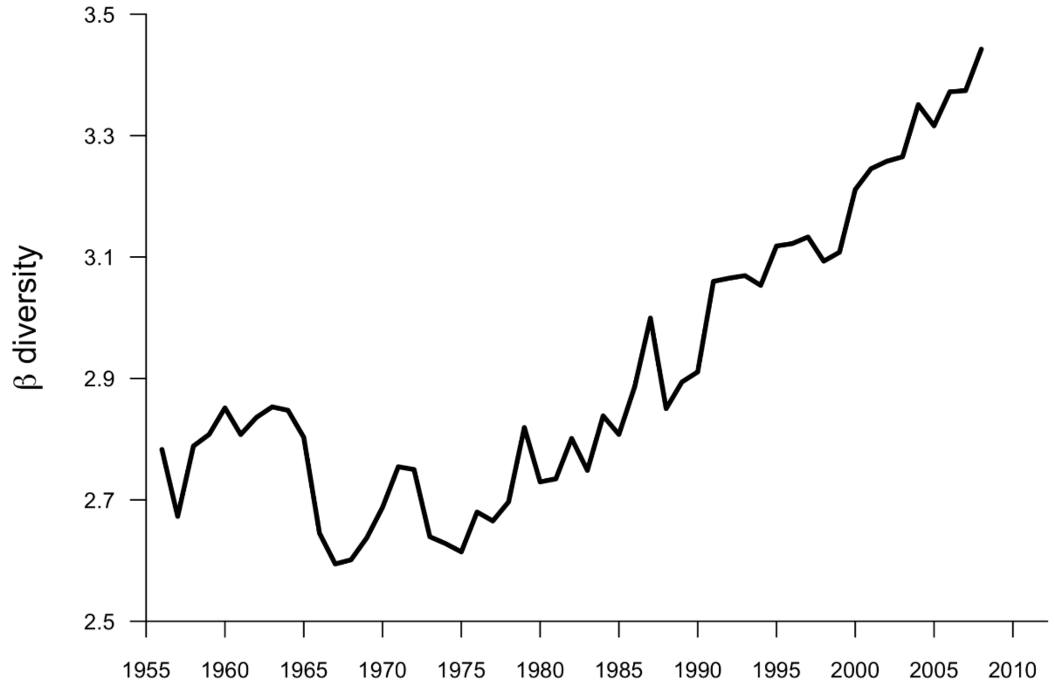

S2 Fig. Crop district-level β-diversity (turnover) in India between 1956 and 2008. β-diversity was calculated for each year by dividing country-level diversity (γ-diversity) by the mean district-level diversity (α-diversity) for the same year: ($\beta = \gamma/\alpha$).
