# Supplementary material for: Agricultural intensification was associated with crop diversification in India (1947-2014)
Source: PLoS One. 2019 Dec 11;14(12):e0225555. doi: 10.1371/journal.pone.0225555 (PMC6905533; doi:10.1371/journal.pone.0225555)
Supplement: S3 Fig — (PDF) [file pone.0225555.s005.pdf]

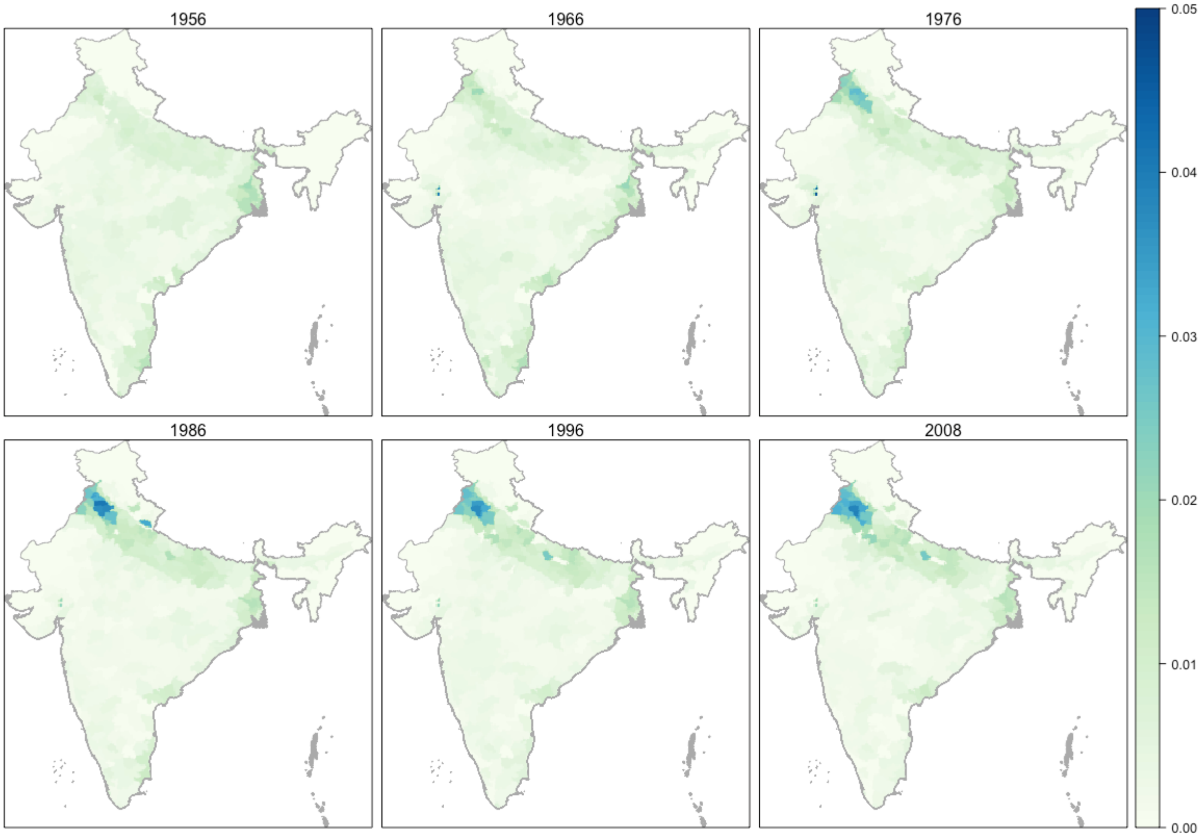

S3 Fig. Cereal production by district, as % of the national area, in India for select years between 1956 and 2008.
