# Supplementary material for: Agricultural intensification was associated with crop diversification in India (1947-2014)
Source: PLoS One. 2019 Dec 11;14(12):e0225555. doi: 10.1371/journal.pone.0225555 (PMC6905533; doi:10.1371/journal.pone.0225555)
Supplement: S4 Fig — (A). Proportional area of selected crops in India in 1956, 1982, and 2008. (B). Proportional area of selected crops in India in 1956, 1982, and 2008. (C). Proportional area of selected crops in India in 1956, 1982, and 2008. (PDF) [file pone.0225555.s006.pdf]

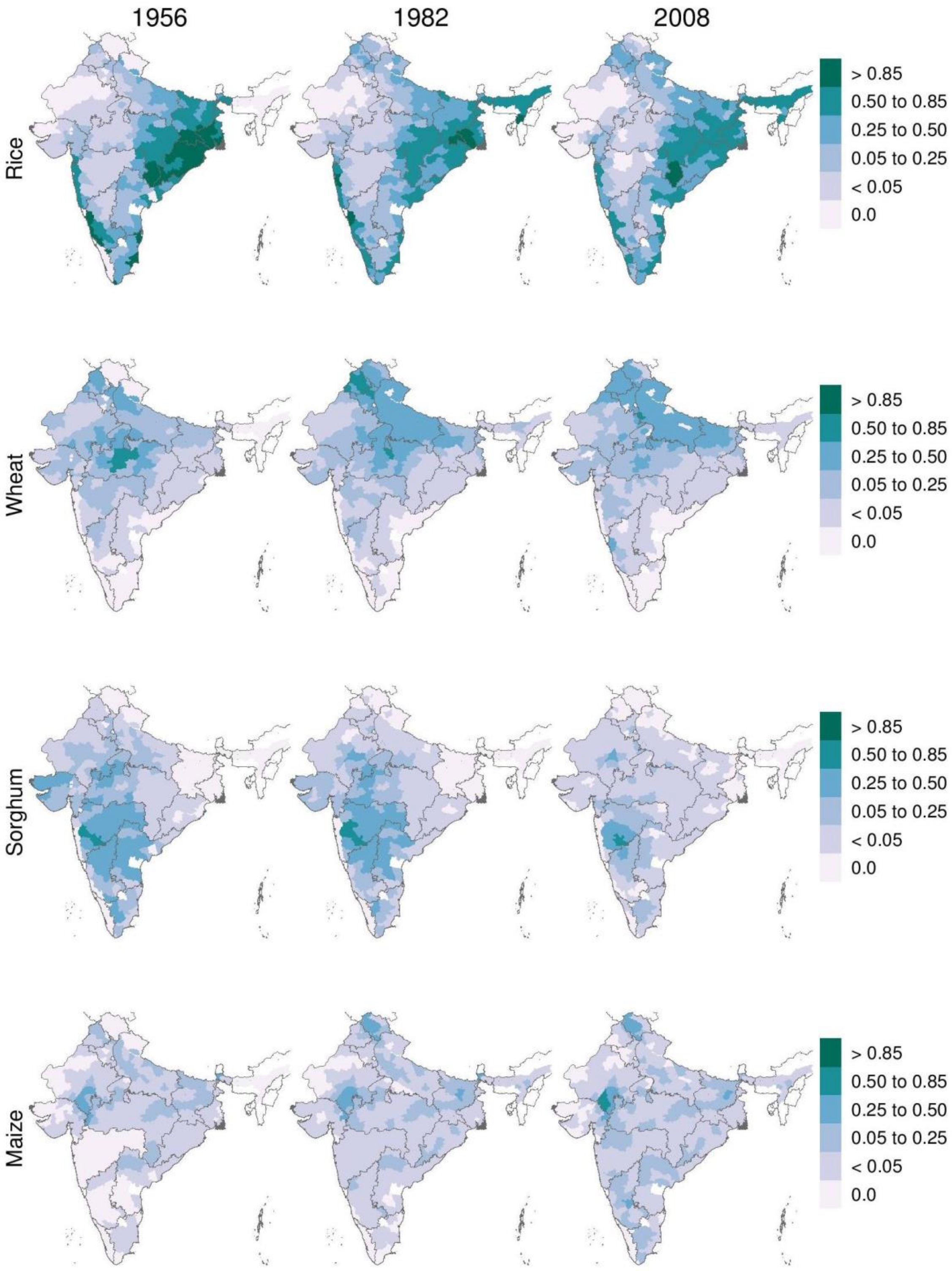

S4 Fig (A). Proportional area of selected crops in India in 1956, 1982, and 2008.

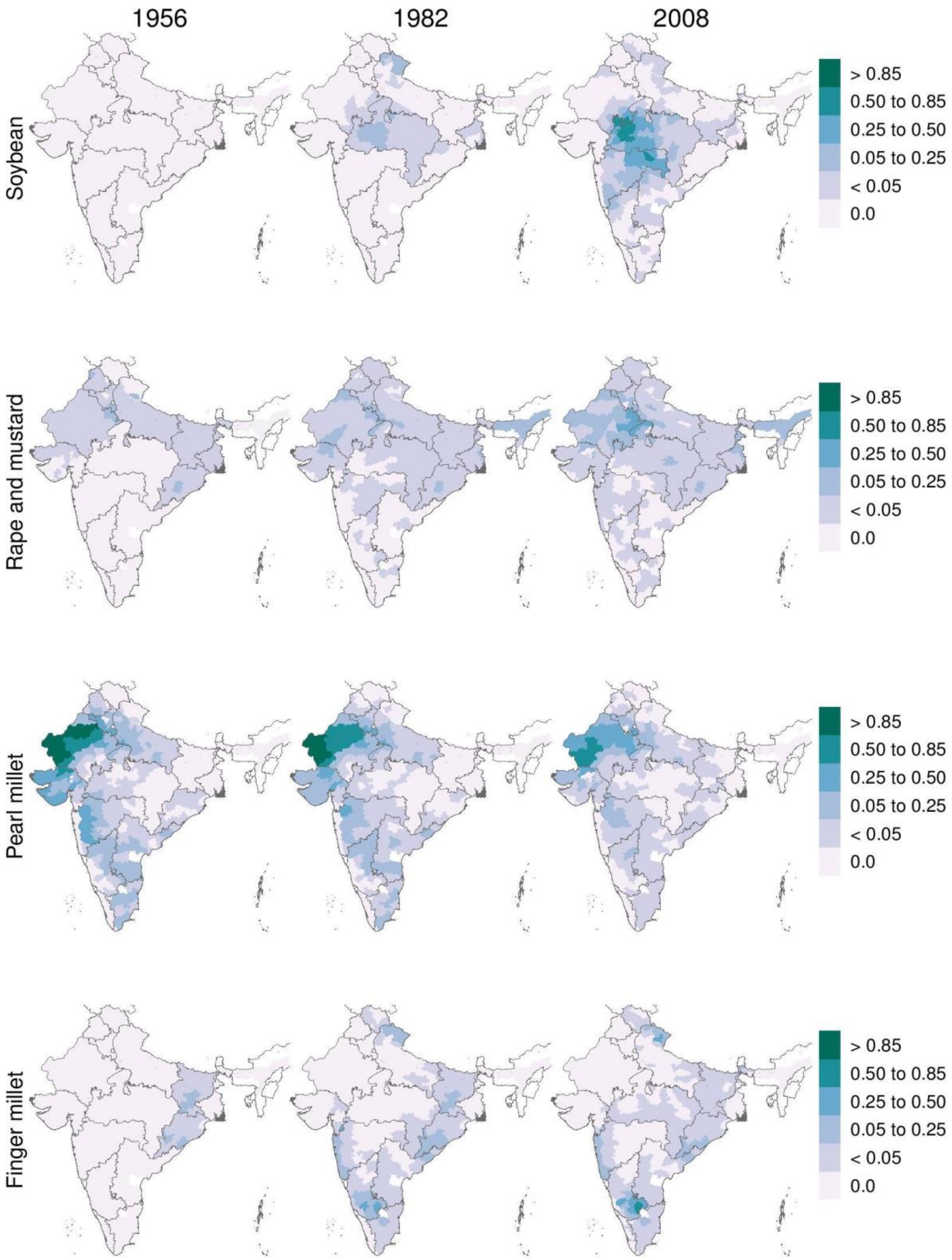

S4 Fig (B). Proportional area of selected crops in India in 1956, 1982, and 2008.

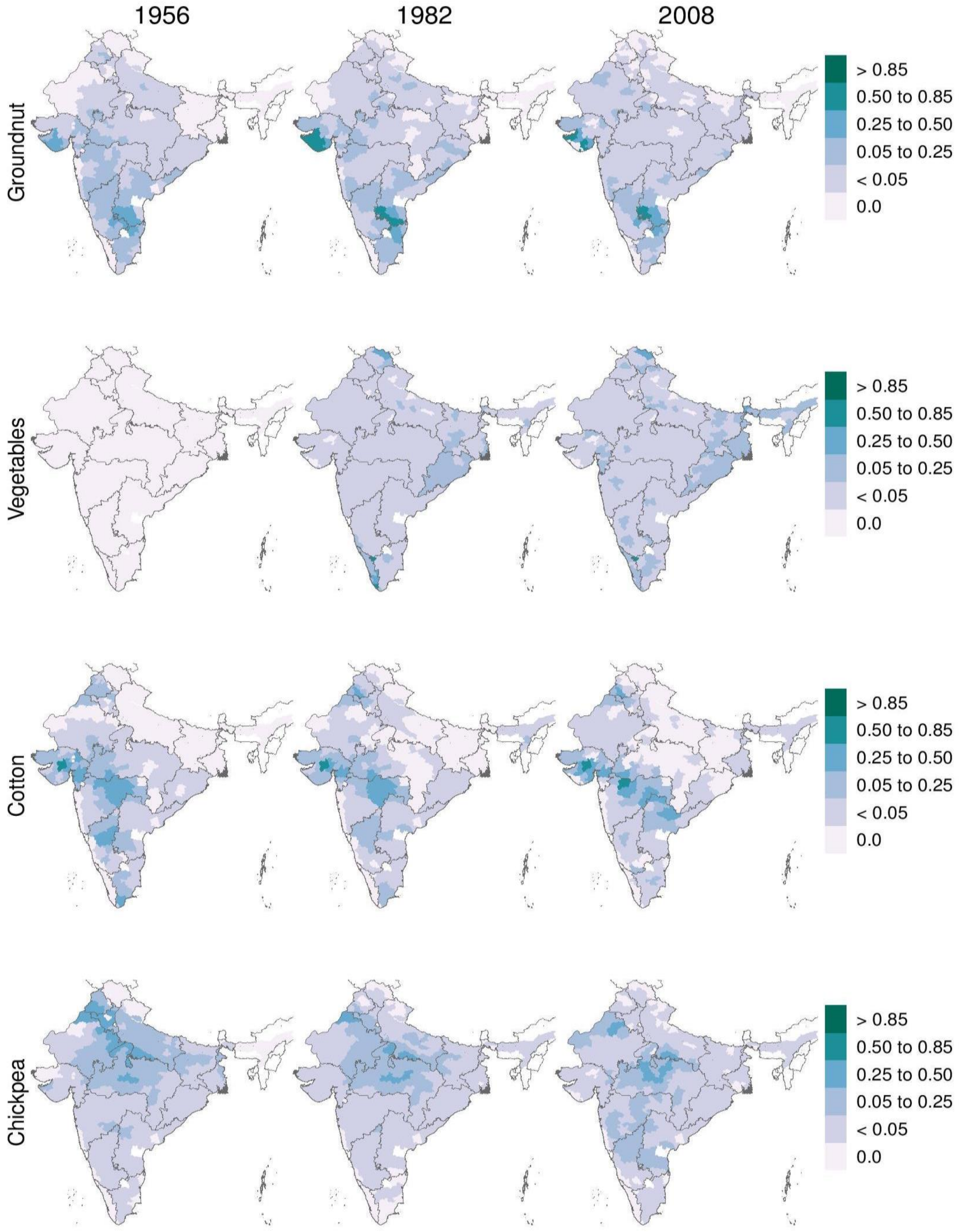

S4 Fig (C). Proportional area of selected crops in India in 1956, 1982, and 2008.
