# Supplementary material for: Agricultural intensification was associated with crop diversification in India (1947-2014)
Source: PLoS One. 2019 Dec 11;14(12):e0225555. doi: 10.1371/journal.pone.0225555 (PMC6905533; doi:10.1371/journal.pone.0225555)
Supplement: S5 Fig — (PDF) [file pone.0225555.s007.pdf]

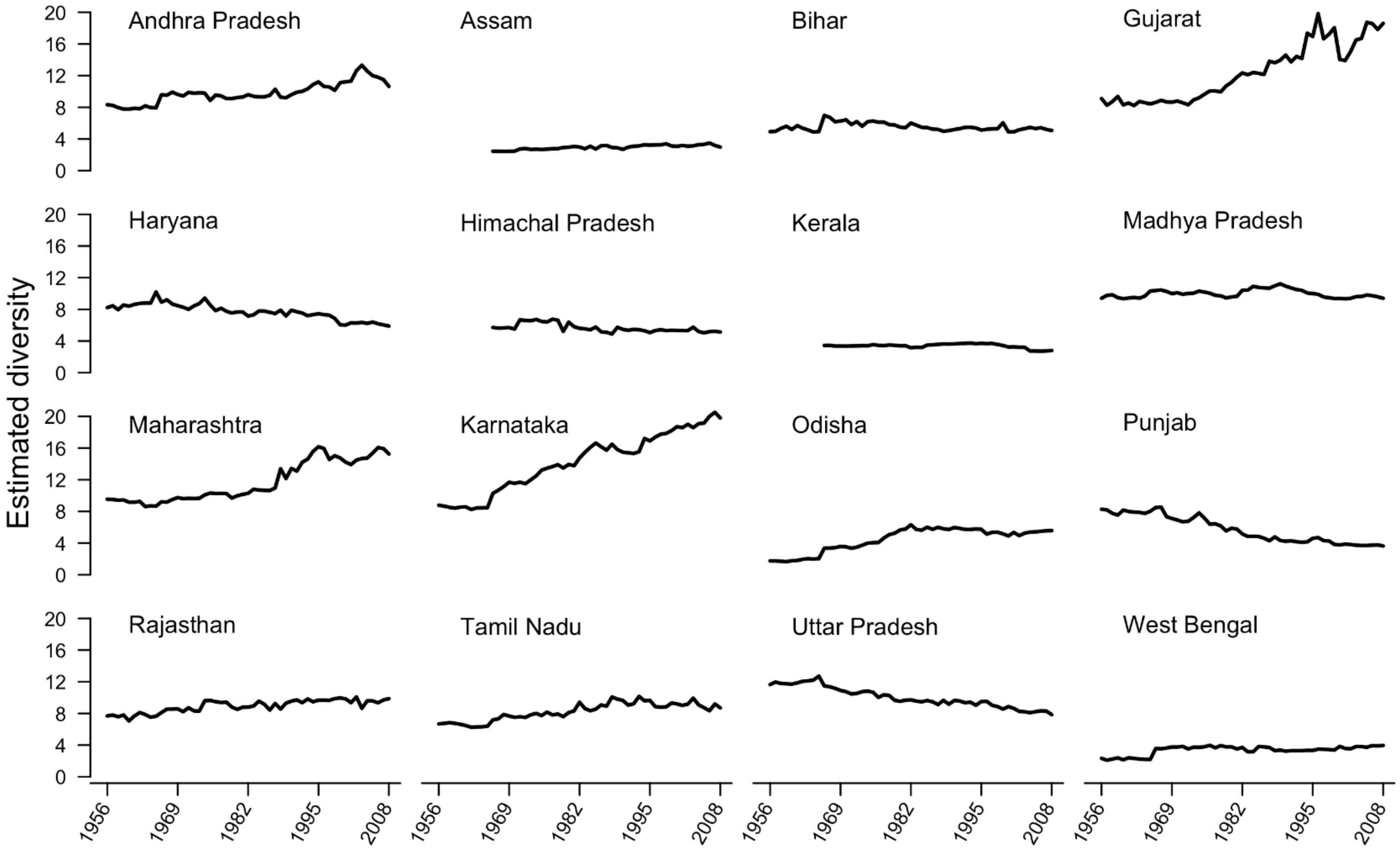

S5 Fig. Crop diversity in the 16 current states (1966 boundaries) included in the district level datasets for the period from 1956 to 2008.
