# Supplementary material for: Agricultural intensification was associated with crop diversification in India (1947-2014)
Source: PLoS One. 2019 Dec 11;14(12):e0225555. doi: 10.1371/journal.pone.0225555 (PMC6905533; doi:10.1371/journal.pone.0225555)
Supplement: S6 Fig — (A) Increase in the proportion of area planted to cereals as a response to increased intensity of cereals. (B) Decline in crop diversity as a response to an increase in the proportion of area planted to cereals. (C) Decline in crop diversity in response to an increase in cereal yields. (PDF) [file pone.0225555.s008.pdf]

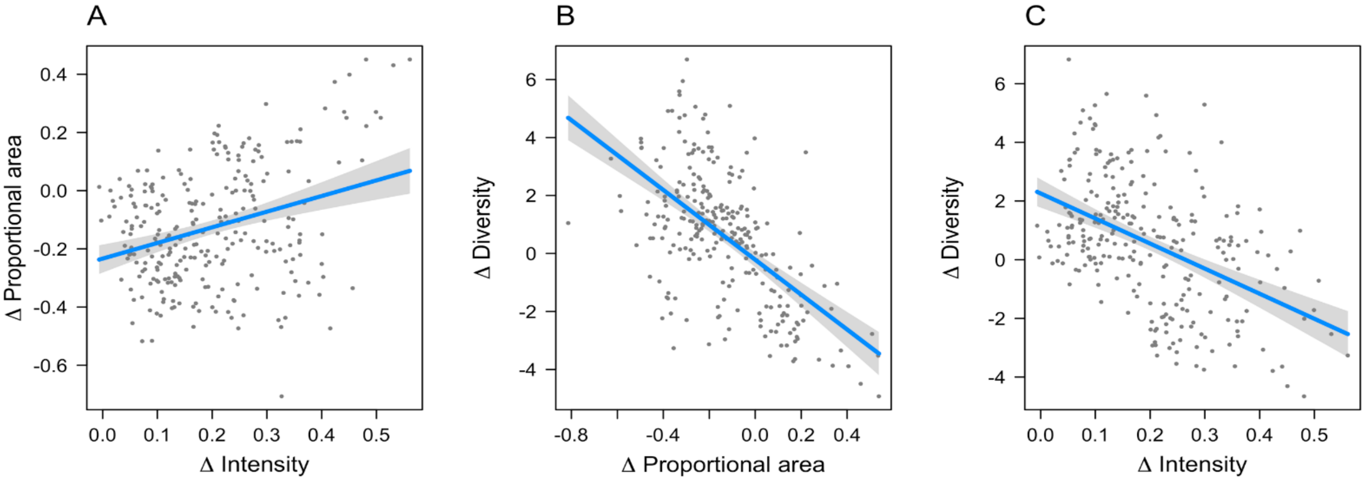

S6 Fig. Regression models fit to district-level data to explain changes in crop diversity in India between 1956-2008 as a response to increased intensification. (A) Increase in the proportion of area planted to cereals as a response to increased intensity of cereals. (B) Decline in crop diversity as a response to an increase in the proportion of area planted to cereals. (C) Decline in crop diversity in response to an increase in cereal yields.
